# From Strikers to Keepers: Somatotype of Football Players from Slovakia

**DOI:** 10.3390/sports12100271

**Published:** 2024-10-09

**Authors:** Branislav Kolena, Barbora Šviríková, Mária Vondráková

**Affiliations:** Department of Zoology and Anthropology, Faculty of Natural Sciences and Informatics, Constantine the Philosopher University, Nabrezie Mladeze 91, 949 01 Nitra, Slovakia

**Keywords:** somatotype, football players, player position, sport

## Abstract

Background/Objectives: This study aims to analyze and compare the somatotypes of professional football players from Slovakia with a non-athletic population. Methods: Comparative analysis of professional soccer players by their positions, goalkeepers (n = 4; 8%), defenders (n = 16; 32%), strikers (n = 15; 30%), and midfielders (n = 15; 30%), in average age 16.88 ± 1.29 years, based on selected anthropometric parameters, somatotype components, and the resultant somatotype using the Heath–Carter method. Results: The average somatotype of the soccer players was categorized as an ectomorphic mesomorph (40%). Goalkeepers showed significantly greater body height (BH 187.98 ± 3.166 cm) and weight (BW 82.33 ± 4.922 kg) than midfielders (BH 179.25 ± 6.126 cm; *p* = 0.03; BW 68 ± 6.304 kg; *p* = 0.014) and strikers (BH 176.04 ± 4.998 cm; *p* = 0.026; BW 68.93 ± 6.591 kg; *p* = 0.026). Defenders had significantly greater BH (182.14 ± 4.853 cm; *p* = 0.026) than strikers. Goalkeepers also had a significantly higher BMI than midfielders (23.28 ± 0.698 vs. 21.14 ± 1.282 kg/m^2^; *p* = 0.02) and greater epicondylar width of the humerus (EWH 7.36 ± 0.14 cm) compared to strikers (EWH 6.79 ± 0.308 cm; *p* = 0.014). The average somatotype values identified goalkeepers [2.0–4.1–3.1], defenders [1.6–3.9–3.2], and strikers [1.7–4.2–2.9] as ectomorphic mesomorphs, while midfielders were identified as mesomorph–ectomorphs [1.6–3.7–3.6]. Conclusions: The results highlight the importance of somatotype in player position suitability and its impact on physical attributes in football.

## 1. Introduction

Somatotype plays a crucial role in sports science and athletic performance enhancement by aiding in athlete selection, training program design, and performance prediction. Studies have shown that somatotype can influence sport-specific explosive variables like vertical jump, sprinting speed, and power-to-body-mass ratio [1]. Additionally, somatotype components have been linked to various performance-related parameters in sports such as running speed, agility, and maximum aerobic power in disciplines like handball [2]. Furthermore, somatotype assessments have been utilized to identify talent, guide training programs, and avoid errors in selecting athletes for specific sports, especially during the puberty period when significant changes in somatotype occur [3]. The application of artificial neural networks in somatotype determination has shown promising results, providing accurate ratings for endomorphy and mesomorphy in young women, which can be beneficial for personalized training and performance optimization [4].

The somatotype of football players varies depending on the specific requirements of the sport and the position played. Research on elite Italian soccer players revealed an ecto-mesomorphic somatotype, with a reduction in the endomorphic component over the years [5]. Female handball players, particularly those in the Serbian national team, predominantly exhibit a mesomorph somatotype, attributed to the physical demands of the sport and individual muscle development [6]. Mexican professional footballers showed a prevalence of balanced mesomorph somatotype, correlating with strength, explosive strength, and speed capacities [7]. In junior elite handball players, endomorphy and ectomorphy were associated with poorer performance in various physical tests, while mesomorphy predicted worse standing long jump performance [4]. Additionally, university female soccer players in Mexico displayed an endo-mesomorphic somatotype, with a dominant relative adiposity component potentially influencing their athletic performance [8].

Ideal body characteristics for football players encompass a combination of anthropometric and physiological factors. Research indicates that regular football practice leads to favorable anthropometric and biochemical profiles, including lower body fat percentages and balanced glucose and lipid levels [9]. Additionally, a study on elite male football players aged 13–15 suggests that early-maturing players exhibit better physical performance in terms of height, sprint speed, and muscular power, while late-maturing players show significant growth in physical function, particularly in height and muscular power, over time [10]. Furthermore, a comparative analysis of two generations of football players highlights the importance of morphological characteristics, with successful players having increased body height, ideal body weight, muscle components, and lower body fat percentages [11]. These findings emphasize the significance of balanced body composition, muscular strength, and appropriate physiological parameters for optimal football performance.

This study aims to comprehensively analyze the anthropometric characteristics of young male football players in Slovakia, focusing on how these measurements vary by playing position.

## 2. Materials and Methods

This study involved professional players from the sports club MŠK Žilina, Slovakia (n = 50), under the supervision of a physiotherapist. The control group consisted of individuals from the general male population from the same region (n = 50; average age 16.82 ± 1.19; BMI 22.73 ± 3.828 kg/m^2^; more detail in Supplementary Table S1). The recruitment of participants and data collection occurred between August and September 2023. Inclusion criteria to enroll participants in this cross-sectional observational study included good health with no presence of diseases, not being users of tobacco products or other substances, age between 18 and 30 years, and Slovak origin. For professional football players, they must be professional members of a sports club. Participants in the control had regular physical activity for at least 2 years. Participants not meeting the above criteria were excluded. All participants were dressed in underwear to ensure the accurate and unobstructed measurement of body dimensions. Measurements took place in the morning at 08:00, prior to any training or physical activity to avoid the influence of exercise on body composition. The measurements were conducted in a room with a controlled temperature of 20 °C, providing a consistent and neutral environment. Data were gathered by observing participants without influencing their actions. The participants were positioned in the reference plane *basis dorsalis*, ensuring uniform posture and body alignment. All measurements were taken using calibrated instruments to guarantee precision and reliability. Each measurement was performed three times, and the results were averaged to account for any potential variation. A trained professional conducted all measurements to maintain consistency and minimize inter-operator variability.

Participants were informed about the procedure and conduct of the research before undergoing anthropometric measurements. They participated voluntarily, anonymously, without financial compensation, and only after confirming informed consent. The study was conducted in accordance with the Declaration of Helsinki and approved by the Ethics Committee of Constantine the Philosopher University in Nitra, Slovakia (UKF-2022/1191-2:191013).

### 2.1. Anthropometry

All anthropometric measures were performed by a trained researcher using a standardized protocol. The parameters for determining the somatotype were recorded using standard anthropometric techniques. Measurements were taken on the dominant side of the body and included the following: body height (cm); body weight (kg); thickness of four skinfolds (TS—triceps skinfold, SsS—subscapular skinfold, SiS—suprailiac skinfold, CS—calf skinfold; mm); bone dimensions (EWH epicondylar width of the humerus and EWF—epicondylar width of the femur; cm); and circumferential measurements (BC—circumference of the contracted biceps, CC—circumference of the calf; cm). Height, weight, waist, circumference of the contracted biceps, and circumference of the calf were measured to the nearest 0.1 kg and 0.5 cm, and skin fold was measured to the nearest 0,1 mm. Epicondylar width of the humerus and femur was taken using a digital caliper (POWER-FIX PROFI, model Z22855, Owim GMbH a Co. KG, Neckarsulm, Germany) with an accuracy of 0.05 mm. BMI was calculated by dividing an adult’s weight in kilograms by their height in meters squared (kg/m^2^).

The anthropometric somatotype was determined using the Heath–Carter method from 1990 [12,13], using the following equations for endomorphy (EnC), mesomorphy (MeC), and ectomorphy (EcC):

- ENDOMORPHY** = −0.7182 + 0.1451 × X − 0.00068 × X^2^ + 0.0000014 × X^3^, where X = (170.18/height in cm) × (TS + SaS + SiS in mm).
- MESOMORPHY** = (0.858 × EWH) + (0.601 × EWF) + (0.188 × BC) + (0.161 × CC) − (0.131 × height) + 4.5.
- ECTOMORPHY** was determined using the height–weight ratio, which is the ratio of height in cm to the cube root of body weight in kg. Based on the HWR value, the ectomorphic component was calculated using one of the three equations: [12,13].
- HWR = height/weight^1/3.^
- If HWR ≥ 40.75, then ectomorphy = 0.732 × HWR − 28.58.
- If HWR < 40.75 and > 38.25, then ectomorphy = 0.463 × HWR − 17.63.
- If HWR ≤ 38.25, then ectomorphy = 0.1.

To visualize the 2D somatotype in a somatograph, we used software shklyn/somatotype https://shklyn.github.io/somatotype/?fbclid=IwAR2AgCiHw3PN3FQ7Y1Zfh78InG56oRBPn3nKpWAQoG5WcujbxCYJPILQf6A (accessed on 18 July 2024) [14] by entering the values of the endomorphy, mesomorphy, and ectomorphy components.

### 2.2. Statistical Analysis

For the evaluation of the analysis results, we used the JAMOVI Statistical Program (Version 2.3.28). Data normality was tested using the Shapiro–Wilk test. To determine the correlation between the analyzed anthropometric parameters, we used a parametric test—Pearson’s correlation coefficient (if the data had a normal distribution)—or a non-parametric test, Spearman’s correlation coefficient (if the data did not have a normal distribution). Data were log-transformed to base 10 to compare the results between the experimental group of football players and the control group (the Mann–Whitney U test) and to compare the football players based on playing position (the Kruskal–Wallis test).

## 3. Results

Except for age, BC, MeC, and EcC, all values of the analyzed parameters were higher in the control group than in the group of football players. A statistically significant difference was observed in the case of TS, SsS, SiS, Cs, and EnC (≤0.001). Descriptive statistics of the anthropometric parameters of football players (A) and the control group (B) are shown in Table 1.

**Figure 1:**
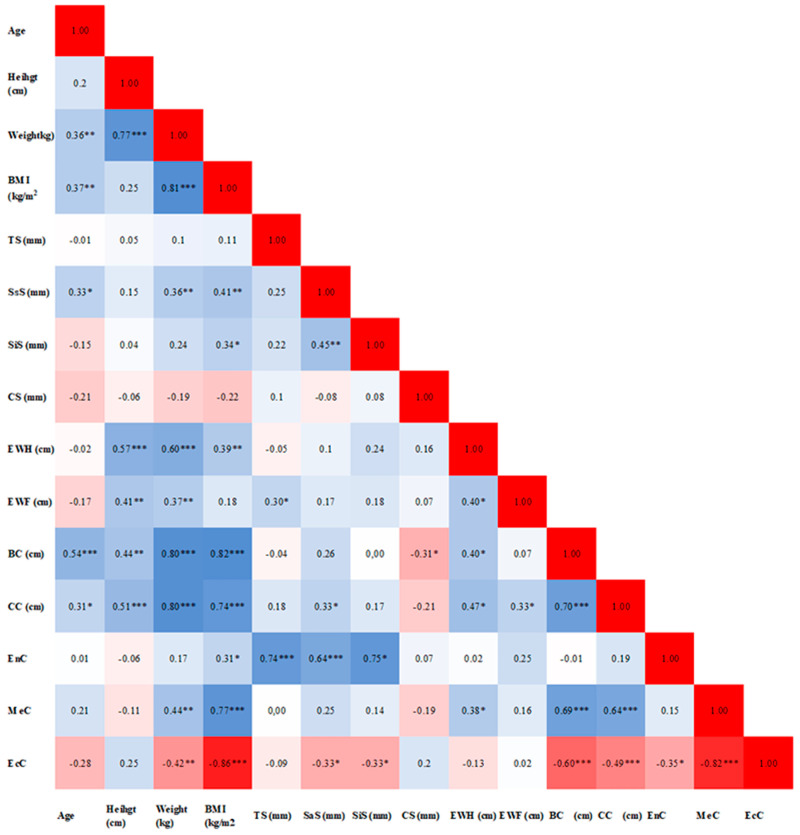
The intercorrelations among the anthropological parameters. Notes: BMI—body mass index; TS—triceps skinfold; SsS—subscapular skinfold; SiS—suprailiac skinfold; CS—calf skinfold; EWH—epicondylar width of the humerus; EWF—epicondylar width of the femur; BC—biceps circumference; CC—calf circumference; EnC—endomorphic component; MeC—mesomorphic component; EcC—ectomorphic component; red color indicates a stronger negative correlation, while the blue color indicates a stronger positive correlation, * *p* < 0.05, ** *p* < 0.01, *** *p* < 0.001.

The intercorrelations among the anthropological parameters in football players are shown in Figure 1.

**Figure 2:**
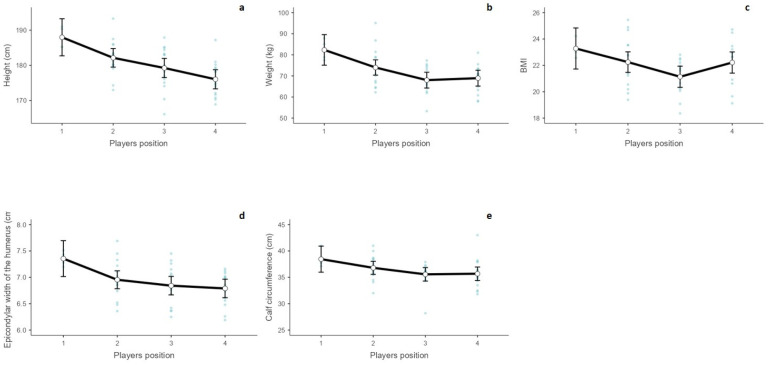
Differences in height (**a**), weight (**b**), BMI (**c**), epicondylar width of the humerus (**d**), and calf circumference (**e**) across the players’ positions (1—goalkeeper, 2—defender, 3—midfielder, 4—striker; blue dots—observed scores).

In Supplementary Table S1, we show the baseline characteristics of football players according to their players’ positions. Using the Kruskal–Wallis test and based on the analysis of multiple comparisons of average ranks, we found statistically significant differences between the anthropometric parameters across the players’ positions, such as (Figure 2a–e) goalkeeper and midfielder positions in terms of body height (*p* = 0.03), weight (*p* = 0.014), BMI (*p* = 0.02), and calf circumference (*p* = 0.039). Additionally, significant differences were observed between the goalkeeper and striker positions for body height (*p* = 0.026), body weight (*p* = 0.026), and epicondylar width of the humerus (*p* = 0.014). Body height also differed significantly when comparing the defender and striker positions (*p* = 0.008).

**Figure 3:**
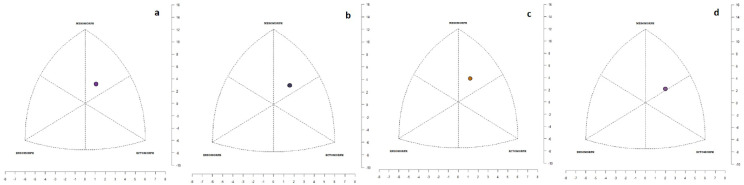
Average somatotype of goalkeepers (**a**), defenders (**b**), strikers (**c**), and midfielders (**d**) visualized by somatograph.

In the entire group, ectomorphic mesomorphs predominated (40%), followed by mesomorphic ectomorphs (20%). Balanced mesomorphs and the mesomorph–ectomorph subcategory each accounted for 18%. The least represented categories were balanced ectomorphs and the central somatotype, each comprising 2%. Based on the mean average values of endomorphy, mesomorphy, and ectomorphy, goalkeepers can be characterized as ectomorphic mesomorphs (2–4.1–3.1; Figure 3a), similar to defenders (1.6–3.9–3.2; Figure 3b) and strikers (1.7–4.2–2.9; Figure 3c). This somatotype category was also observed across the entire group of football players (the exception was midfielders, who were categorized as mesomorphic ectomorphs (1.6–3.7–3.6; Figure 3d).

## 4. Discussion

Body composition assessment is a relevant element not only in the management of athletes but also in the biomedical field, research, and daily practice in the medical and nutritional fields. The equations used in the methodology and the visualization of the somatograph through the mentioned online software can assist both the professional and general public to navigate the given issue better and subsequently seek expert assistance in achieving medical goals. In this study, we address the anthropometry and somatotyping of male football players from the Slovak population aged 15–19 years. Before comparing our results, it is essential to note that variables (such as football league, players’ nationality, and age differences) could affect the validity of this comparison.

Gil et al. [15] conducted a study on young football players aged 14–17 (n = 127) playing for a Spanish football club, finding that their body height and weight were higher compared to a control group, which contradicts our study’s conclusions. Our findings, however, align with a study from 2016 [16] that observed smaller height and lower weight among Macedonian first-league football players aged 17–18 compared to their peers. According to Bandyopadhyay [17], shorter stature in footballers is a disadvantage in achieving optimal ball control during jumps.

In a study from 2019 [18], the authors compared Bosnian first-league football players aged 18–32 (n = 26) with a non-athlete population aged 18–38 (n = 22) and recorded a statistically significant difference in BMI, with the non-athlete group having a significantly higher BMI. We observed a higher BMI in the control group as well, but not at a statistically significant level. According to the WHO [19], based on the average values of individuals from both populations analyzed, they can be classified into the optimal weight category. However, it is important to note that BMI does not distinguish between excess body fat, bone mass, or musculature, nor does it interpret fat distribution [20].

Gontarev et al. [16] analyzed Macedonian first-league football players aged 14–18 (n = 486) and, similar to our study, found that football players had lower BMI values compared to the general Macedonian population (n = 779) aged 14–18. Gil et al. [15] reported that triceps, subscapular, and suprailiac skinfolds were greater in the general population, which corresponds with our findings. Bandyopadhyay [17] compared young football players (n = 46) and volleyball players (n = 82) with a sedentary population (n = 50) from India aged 20–24 years and demonstrated statistically significant differences in all skinfolds mentioned by Gil et al. [15], as well as calf skinfold and circumference, with higher values in the sedentary population. Our findings, except for calf circumference, align well with these studies.

Football requires the development of a robust skeletal structure in the lower limbs, as every football game involves numerous impacts and stretches in the lower extremities [21]. We found no statistically significant difference in the epicondylar dimensions of the humerus and femur between the experimental and control groups. Both parameters had higher average values in the control group, although the differences were minimal. Bandyopadhyay [17] recorded higher average values for Indian football players compared to the general Indian population, but the differences were minimal.

The average somatotype of football players in our study was classified as ectomorphic mesomorph with a reduced endomorphic component. Kaplanová et al. [22] noted that the endomorphic component is the least influenced by heredity and is significantly affected by environmental factors (such as training frequency, intensity, and nutritional value of consumed food), while the mesomorphic component is most influenced by genetics. In our study, the most common somatotype for football players was ectomorphic mesomorph, while the general population was endomorph–ectomorph. Previous studies have confirmed that height and weight are crucial performance factors for football players [22,23]. Taller and heavier players are more suitable for goalkeeper positions, while shorter and lighter players are better suited as midfielders [24]. Players’ heights across various positions typically range from 170 to 190 cm [25]. The average height of the football players analyzed in our study falls within this range.

Cavia et al. [26] observed significant weight differences among professional Spanish football players aged 19–35 (n = 57) between positions, with goalkeepers having the highest body weight. We observed similar results among Slovak professional football players aged 15–19, noting statistically significant weight differences between goalkeepers and midfielders and between goalkeepers and strikers. In the study mentioned above [26], the authors did not record statistically significant height differences, although goalkeepers and defenders were taller than midfielders and strikers, which aligns with our results. We found statistically significant height differences between goalkeepers and strikers, goalkeepers and midfielders, and defenders and strikers.

Fidelix et al. [27] found that professional players from Southern Brazilian football clubs aged 15–17 (n = 67) had taller height and heavier weight for goalkeepers compared to midfielders and strikers, which aligns with our study. This could be because strikers perform more sprints, and midfielders engage in increased running activity at moderate and high speeds during the game [25]. Lago-Peñas et al. [24] divided representative Spanish football players aged 12–19 into six positions: goalkeepers, central defenders, external defenders, central midfielders, external midfielders, and forwards. In contrast to our findings, central defenders were the tallest and heaviest. This discrepancy might be due to different player position classifications. Cavia et al. [26] noted that taller height and greater weight are advantageous for goalkeepers who must compete for aerial balls and cover the goal area to prevent scoring. Taller defenders also benefit from aerial control and space coverage for defense [25].

Our study found a statistically significant BMI difference between goalkeepers and midfielders, with goalkeepers having the highest BMI, consistent with a study from 2019 [26]. Hazir [28] analyzed Turkish Super League players with an average age of 25.7 years (n = 161) and Turkish First League players with an average age of 24.1 years (n = 144) and found no statistically significant BMI differences across positions, possibly due to the lower average age (16.88 years) of the players in our study. In a study from 2014 [27], the authors found that goalkeepers had significantly greater triceps skinfold thickness compared to strikers, but our results did not show significant differences in any skinfold measurements, with goalkeepers showing the highest values in all skinfold thicknesses measured. Our results are consistent with the previously mentioned study [26].

We found that goalkeepers had significantly greater epicondylar width of the humerus than strikers. Cavia et al. [26] did not find significant differences in bone dimensions, suggesting a similar bone structure among football players regardless of position. They also noted no significant differences in biceps and calf circumferences, although goalkeepers had the highest circumferential values, which our study agrees with. There were significant differences in endomorphy between goalkeepers and other positions, and in ectomorphy between defenders and midfielders, but no differences in terms of mesomorphy were observed among young Italian football players aged 12–19 (n = 112) [23]. This differs from our findings, where midfielders had higher ectomorphy compared to forwards, though this was on the threshold of statistical significance. Bandyopadhyay [17] considered mesomorphic and to some extent meso-ectomorphic types as promising football players.

Kaplánová et al. [22] studied Slovak football players aged 19–26 (n = 50) and concluded that goalkeepers were predominantly endomorphic mesomorphs, defenders and strikers balanced mesomorphs, and midfielders had a balanced somatotype. The average somatotype was categorized as balanced mesomorph, consistent with Spanish footballers aged 19–35 (n = 57), with the most frequent somatotype being ectomorphic mesomorph [26]. They also noted positional differences in somatotype: goalkeepers and strikers were balanced mesomorphs, while defenders and midfielders were ectomorphic mesomorphs. In our study, goalkeepers, defenders, and strikers were ectomorphic mesomorphs, except midfielders.

Somatotype components change during adolescence, with endomorphy and ectomorphy decreasing and mesomorphy increasing [29]. Gil et al. [15] also observed a decrease in ectomorphy with age among Spanish footballers aged 14–19 (n = 203). Erceg, Grgantov, and Milié [30] categorized Croatian senior amateur footballers as mesomorph-endomorph and suggested that this body type could reduce performance quality during matches. Goalkeepers in this study were classified as balanced endomorphs. Fidelix et al. [27] classified goalkeepers, strikers, and defenders as balanced mesomorphs, while midfielders were ectomorphic mesomorphs, with the average player somatotype being balanced mesomorph. In our study, midfielders also fell into a different somatotype category, consistent with their greater distance covered during games compared to strikers and defenders [27].

Hazir [28] identified the average somatotype of Turkish Super League footballers as balanced mesomorph. Kaplánová et al. [22] argue that it has not yet been conclusively proven that somatotypology is consistent across different countries, even though Fidelix et al. [27] consider the balanced mesomorph somatotype to be ideal for football players. On the other hand, Psotta [25] asserts that due to the physical demands of modern football, such as sprinting, changing direction, and pivoting, individuals with a higher ectomorphic component (slimness) and relatively lower mesomorphic component (muscularity) are favored. From the above, it follows that there is no single ideal prototype for a football player. However, determining an individual’s somatotype can help select the most suitable position for the player [31]. Since body proportions among Europeans are highly variable [32], this genetic aspect may be the cause of the differing somatotypes observed in the comparative studies mentioned above.

While somatotype provides valuable insights for talent development, it is essential to consider individual variability and the dynamic nature of body composition throughout an athlete’s career. The practical application of somatotype and morphology in talent development and injury risk assessment is also significant in sports. Understanding an athlete’s body composition can guide effective training and selection processes, ultimately enhancing performance and reducing injury risks. Somatotype profiles can inform talent selection, as certain body types correlate with specific athletic abilities. For instance, mesomorphic children showed superior performance in strength tests [33]. Training can modify somatotype, enhancing athletes’ suitability for particular sports, as demonstrated in middle-distance runners [3]. Body morphology influences injury risk, particularly in critical care settings, where specific body shapes and BMI correlate with pressure injury risks [34]. In sports, understanding somatotype can help identify athletes at risk of injuries due to biomechanical inefficiencies related to their body composition [4].

Our study provides insights into the somatotype of professional football players from Slovakia, a region where comparable research is currently lacking. Our findings therefore fill an existing gap in the literature on the somatotype of football players globally. By contributing this regional perspective, our study enhances the overall understanding of the physical characteristics of athletes in the sport across different countries. The main findings of this study reveal that young Slovak football players exhibit a predominantly ectomorphic–mesomorph somatotype, with significant differences in anthropometric parameters compared to the general population, particularly in skinfold thicknesses and the endomorphic component. This suggests that body composition and somatotype are influenced by the demands of football training, with certain somatotypes potentially being more advantageous for specific positions. This study’s relevance lies in its contribution to understanding the physical profiles of young athletes, providing insights into how somatotype can be used as an objective criterion for talent identification and player selection. Practically, the findings could be applied by coaches and sports scientists to optimize athlete development programs by matching players to positions that align with their somatotype, particularly in the early stages of athletic development. This research has implications for refining selection criteria in youth football and could be integrated into training regimens that consider an athlete’s morphological predisposition for specific roles on the field, ultimately enhancing performance at higher competitive levels.

While providing valuable insights into the anthropometry and somatotype of male football players from the Slovak population aged 15–19 years, our study has several limitations.

### Strengths and Weaknesses of the Study

While providing valuable insights into the anthropometry and somatotype of male football players from the Slovak population aged 15–19 years, our study has several limitations. The small sample size, specific to Slovak football players, limits generalizability to other populations and age groups. Focusing on a narrow age range may not capture the full spectrum of developmental changes. As a cross-sectional study, it cannot infer longitudinal changes or causal relationships between training, somatotype, and performance. Variations in training regimens, nutritional intake, and environmental factors are not extensively accounted for, which could influence the results. The relatively small sample size for each position limits the statistical power to detect significant differences. The homogeneous study population lacks genetic diversity, affecting applicability to diverse groups. Additionally, the absence of direct measures of football performance makes it challenging to correlate anthropometric and somatotype characteristics with on-field performance outcomes. One of the limitations of this study is the use of the Heath–Carter method, which may not correspond with somatotype determination methods used in other research. However, we still consider the comparison of somatotype to be accurate. Our study has several strengths, including the use of standardized anthropometric techniques, skilled personnel for measurements, and a control group for comparison with the experimental group of football players. The application of the Heath–Carter method and statistical tests further support the validity of the findings. However, this study has limitations, such as a small sample size and a homogeneous population, reducing its generalizability. The cross-sectional design limits the ability to track somatotype changes over time, and the lack of direct performance metrics makes it difficult to correlate anthropometric data with on-field football performance. Additionally, while different player positions were analyzed, the small sample size within each group limits the detection of significant differences. This study also fails to consider external factors like diet, training intensity, and genetic influences, which could impact body composition. To improve future research, increasing the sample size, employing a longitudinal design, and incorporating performance metrics would provide more comprehensive insights. Broadening the age range, controlling for external variables like training regimens and nutrition, and including more diverse populations would enhance the applicability of the findings. Future studies should also explore the genetic and environmental interactions affecting somatotype, offering a more holistic understanding of its role in athletic performance. This study fails to address the considerable variability in soccer performance attributable to factors beyond body morphology, including technical proficiency, tactical understanding, psychological characteristics, and training history. Specifically, innate abilities and cognitive skills may play a more pronounced role at higher levels of competition.

## 5. Conclusions

Our study provides valuable insights into the anthropometric characteristics and somatotypes of young Slovak football players, revealing significant positional differences and aligning with broader trends observed in football populations. Despite its limitations, it underscores the importance of somatotype in player position suitability and the potential influence of genetic and environmental factors on physical attributes in football. Body composition evaluation plays a crucial role not only in athlete management but also in biomedical research and everyday medical and nutritional practices. The formulas employed in the methodology, combined with the somatograph visualization through the provided online software, can aid both experts and the general population in grasping the subject more effectively and seeking specialized support to reach health-related goals.

## Figures and Tables

**Table 1:** Baseline characteristics of the participants.

								Percentiles		
Parameter	Group	n	Mean	Median	SD	Min	Max	25th	75th	*p*
Age	A	50	16.88	17	1.288	15	19	16	18	0.829
	B	50	16.82	17	1.19	15	19	16	18	
Height (cm)	A	50	179.91	180.2	6.112	166.1	193.3	174.95	184.18	0.737
	B	50	180.69	180.05	7.661	166.9	195.5	175.13	185.9	
Weight (kg)	A	50	71.34	71	8.129	53.3	95.1	65.67	75.33	0.497
	B	50	74.07	71.75	12.15	53	112.8	65.25	79.38	
BMI	A	50	21.99	22.17	1.626	18.356	25.45	20.95	22.9	0.738
	B	50	22.73	22.32	3.828	16.512	35.48	20.45	23.52	
TS (mm)	A	50	6.66	6.65	2.084	3.3	11	5	8	≤0.001
	B	50	11.7	10.65	4.1	5.3	22	9	14.93	
SsS (mm)	A	50	6.37	6	1.206	4	10.1	5.6	7	≤0.001
	B	50	9.78	9.15	3.981	5	24.6	7.08	10.3	
SiS (mm)	A	50	5.87	5.5	2.138	3	13.7	4.3	7.3	≤0.001
	B	50	11.26	10	5.486	4.3	28	7.08	13.82	
CS (mm)	A	50	7.03	7.3	2.068	2.3	10.7	6	8.23	≤0.001
	B	50	12.3	11.5	3.714	7	24.7	9.3	15	
EWH (cm)	A	50	6.9	6.94	0.36	6.19	7.69	6.7	7.14	0.607
	B	50	6.93	6.94	0.461	5.74	7.75	6.65	7.22	
EWF (cm)	A	50	9.08	9.07	0.293	8.35	9.82	8.92	9.25	0.978
	B	50	9.13	9	0.49	8.21	10.82	8.83	9.37	
BC (cm)	A	50	32.08	32	2.77	27.5	38	29.38	34	0.751
	B	50	32.03	31.25	3.54	25.2	41.5	29.52	34.55	
CC (cm)	A	50	36.23	36.15	2.528	28.2	43	35	37.85	0.157
	B	50	37.27	36.95	3.304	31.1	46.5	35	38.92	
EnC	A	50	1.66	1.61	0.456	0.943	3.07	1.34	1.92	≤0.001
	B	50	3.09	2.88	1.17	1.587	6.87	2.23	3.6	
MeC	A	50	3.94	3.9	0.871	2.007	6.23	3.49	4.32	0.707
	B	50	3.87	3.97	1.426	1.243	7.99	2.93	4.77	
EcC	A	50	3.23	3.22	0.782	1.516	4.95	2.79	3.58	0.707
	B	50	3.16	3.1	1.571	0.1	7.09	1.97	3.94	

Notes: BMI—body mass index; TS—triceps skinfold; SsS—subscapular skinfold; SiS—suprailiac skinfold; CS—calf skinfold; EWH—epicondylar width of the humerus; EWF—epicondylar width of the femur; BC—biceps circumference; CC—calf circumference; EnC—endomorphic component; MeC—mesomorphic component; EcC—ectomorphic component; SD—standard deviation; Min—minimum; Max—maximum; statistically significant *p*-values are in bold.

## Data Availability

The data that support the findings of this study are available from the corresponding author upon reasonable request.

## Supplementary Materials

The following supporting information can be downloaded at: https://www.mdpi.com/article/10.3390/sports12100271/s1, Table S1: Baseline characteristics of control group and football players according to their players’ positions.
