# Supplementary material for: From Strikers to Keepers: Somatotype of Football Players from Slovakia
Source: Sports (Basel). 2024 Oct 9;12(10):271. doi: 10.3390/sports12100271 (PMC11511079; doi:10.3390/sports12100271)
Supplement: Supplementary file 1 [file sports-12-00271-s001.zip › sports-3221028-supplementary.pdf]

Supplementary Table S1. Baseline characteristics of control group and football players according to their players' positions.

Parameter	Players' position	n	Mean	Median	SD	Min	Max
Age	control group	50	16.82	17	1.19	15	19
	1	4	16.25	16	0.5	16	17
	2	16	17.06	17	1.34	15	19
	3	15	17	17	1.363	15	19
	4	15	16.73	17	1.335	15	19
Height (cm)	control group	50	180.69	180.05	7.661	166.9	195.5
	1	4	187.98	187.85	3.166	185.2	191.03
	2	16	182.14	182	4.853	173	193.3
	3	15	179.25	180	6.129	166.1	187.9
	4	15	176.04	174.9	4.998	168.9	187.2
Weight (kg)	control group	50	74.07	71.75	12.15	53	112.8
	1	4	82.33	82	4.922	77.5	87.8
	2	16	73.99	73.55	8.759	62.2	95.1
	3	15	68	67.7	6.304	53.3	77.3
	4	15	68.93	70.7	6.591	57.9	81
BMI	control group	50	22.73	22.32	3.828	16.512	35.48
	1	4	23.28	23.16	0.698	22.571	24.22
	2	16	22.25	22.13	1.834	19.391	25.45
	3	15	21.14	21.19	1.282	18.356	22.81
	4	15	22.21	22.26	1.586	19.124	24.72
TS (mm)	control group	50	11.7	10.65	4.1	5.3	22
	1	4	7.22	7.15	3.025	3.6	11
	2	16	6.26	5.65	1.953	4	9.3
	3	15	6.63	6.3	1.996	4	10.3
	4	15	6.96	7	2.191	3.3	11
SsS (mm)	control group	50	9.78	9.15	3.981	5	24.6
	1	4	7.05	6.8	1.723	5.6	9
	2	16	6.45	6.3	0.735	5.3	8
	3	15	6.21	6	1.235	4	9
	4	15	6.25	6	1.471	4	10.1
SiS (mm)	control group	50	11.26	10	5.486	4.3	28
	1	4	8.5	8.15	4.041	4	13.7
	2	16	6.23	5.85	1.896	3.3	10.3
	3	15	5.13	4.3	1.608	3.3	9

	4	15	5.52	6	1.797	3	8
CS (mm)	control group	50	12.3	11.5	3.714	7	24.7
	1	4	7.67	7.5	2.371	5	10.7
	2	16	6.64	7.15	2.506	2.7	10.3
	3	15	7.24	7.3	1.77	3.7	10.7
	4	15	7.07	7.3	1.887	2.3	9.3
EWH (cm)	control group	50	6.93	6.94	0.461	5.74	7.75
	1	4	7.36	7.36	0.141	7.2	7.51
	2	16	6.95	6.94	0.355	6.36	7.69
	3	15	6.84	6.85	0.376	6.25	7.45
	4	15	6.79	6.86	0.308	6.19	7.16
EWF (cm)	control group	50	9.13	9	0.49	8.21	10.82
	1	4	9.16	9.17	0.348	8.74	9.58
	2	16	9.15	9.22	0.288	8.64	9.82
	3	15	9.06	9.07	0.272	8.49	9.7
	4	15	9.01	8.98	0.313	8.35	9.54
BC (cm)	control group	50	32.03	31.25	3.54	25.2	41.5
	1	4	34.64	34.75	1.045	33.5	35.55
	2	16	32.4	32.45	3.084	28	38
	3	15	31.07	31	2.461	27.5	36
	4	15	32.07	32.2	2.699	27.8	36.5
CC (cm)	control group	50	37.27	36.95	3.304	31.1	46.5
	1	4	38.45	37.85	1.741	37.1	41
	2	16	36.8	36.6	2.319	32	41
	3	15	35.57	36.1	2.253	28.2	37.9
	4	15	35.68	35.1	2.881	31.8	43
EnC	control group	50	3.09	2.88	1.17	1.587	6.87
	1	4	1.96	1.92	0.882	0.943	3.07
	2	16	1.64	1.67	0.371	1.14	2.36
	3	15	1.57	1.54	0.357	1.009	2.25
	4	15	1.69	1.6	0.501	0.987	2.62

MeC	control group	50	3.87	3.97	1.426	1.243	7.99
	1	4	4.14	4.2	0.436	3.584	4.57
	2	16	3.9	3.81	0.954	2.07	6.23
	3	15	3.66	3.9	0.825	2.007	4.9
	4	15	4.21	3.85	0.885	3.223	5.99
EcC	control group	50	3.16	3.1	1.571	0.1	7.09
	1	4	3.06	3.12	0.211	2.778	3.23
	2	16	3.24	3.25	0.797	1.516	4.51
	3	15	3.6	3.5	0.742	2.156	4.95
	4	15	2.89	2.83	0.786	1.525	4.34
Somatotype	control group	50	7.72	6.5	4.371	1	13
	1	4	2	2	0.816	1	3
	2	16	3.31	3	2.774	1	13
	3	15	2.8	3	1.082	1	5
	4	15	2.07	2	1.1	1	4

Notes: Players' position 1 – Goalkeeper, 2 – Defender, 3 - Midfielder, 4 – Striker; BMI – Body Mass Index; TS – Triceps skinfold; SsS – Subscapular skinfold; SiS – Suprailiac skinfold; CS - Calf skinfold; EWH – Epicondylar width of the humerus; EWF – Epicondylar width of the femur; BC – Biceps circumference; CC – Calf circumference; EnC – Endomorphic component; MeC – Mesomorphic component; EcC – Ectomorphic component; SD – Standard deviation; Min – Minimum; Max – Maximum;
